# Diversity in warning coloration is easily recognized by avian predators

**DOI:** 10.1111/jeb.13074

**Published:** 2017-04-21

**Authors:** L. M. Arenas, M. Stevens

**Affiliations:** ^1^ Department of Zoology University of Cambridge Cambridge UK; ^2^ Centre for Ecology & Conservation College of Life & Environmental Sciences University of Exeter Penryn Cornwall UK

**Keywords:** aposematism, contrast, diversity, ladybird, vision

## Abstract

Warning coloration is a widespread strategy to alert predators about prey unprofitability. The success of this strategy partly depends on predators being able to learn and recognize certain signals as indicators of toxicity, and theory predicts that this is easier if signals converge on similar colours. However, the diversity in warning signal form is astonishing, contradicting predictions. Here, we quantified ladybird signal diversity with respect to avian vision, measuring how unique and discernible each signal is from one another. In addition, we measured signal conspicuousness against a series of backgrounds, namely an average green, average brown, and where we collected each species, to determine whether signals are more contrasting against the ladybirds’ local substrates than compared to average ones. This allowed us to establish whether there are local adaptations in conspicuousness that promote signal diversity. We found that while ladybird signals are unique and recognizable, specialist species are more contrasting against the background they are most commonly found on. However, overall our study suggests that warning signals have evolved to be effective against a wide range of natural backgrounds, partly explaining the success of this strategy in nature.

## Introduction

Animal coloration is often used as a means to avoid predation (Endler, 1978; Stevens, 2013, 2016). Here, the visibility of a colour may determine the prey's survival and ultimately its ability to pass genetic information to the next generation (Mappes *et al*., 2005). One way in which animals can fend off predators is by displaying warning coloration, where bright, conspicuous colour patterns are used to inform potential predators about a prey's unprofitability [aposematism (Poulton, 1890; Cott, 1940; Stevens & Ruxton, 2012; Skelhorn *et al*., 2016)]. In addition, colour patterns should evolve under selection to match conditions of the environment against which they will be displayed (Endler, 1978). This is because visibility will be affected by characteristics such as the contrast of the signal against the background, the environmental light conditions, and the place and substrate where the signal is being displayed (e.g. Arenas *et al*., 2014; Hall *et al*., 2016). Furthermore, physiological properties of the predator's visual system can influence how a signal is perceived and interpreted (Endler, 1978; Guilford & Dawkins, 1991; Endler & Mielke, 2005; Stevens, 2011, 2013). However, only a few studies have empirically established how diverse warning signals are to the eyes of a potential predator (Cortesi & Cheney, 2010; Maan & Cummings, 2012).

The survival of an individual displaying warning colours depends in part on the predator's memory and ability to discriminate between edible and inedible prey (Speed & Turner, 1999; Lynn, 2005; Skelhorn & Rowe, 2006). As such, it would seem appropriate for warning signals to have as little variation as possible, aiding the process of recognition and memory of potential predators (Servedio, 2000). Theoretical studies on colour signalling predict that predator selection will work in favour of maintaining a monomorphic signal, a process known as homogenizing selection (Speed & Ruxton, 2007; Chouteau & Angers, 2012). However, simply visually inspecting the colours of aposematic species it is easy to see that the variation in signal colour and form is impressive, both within (Maan & Cummings, 2012; Stuckert *et al*., 2014) and even among (Summers & Clough, 2001; Arenas *et al*., 2015) species.

Diverse signal forms can reduce the chances of a predator successively encountering the same prey appearances, decreasing the probability of predators becoming familiar with a specific pattern (Mappes *et al*., 2005). This, in turn, may reduce a predator's optimal foraging strategies and decrease attention on a specific pattern. Importantly, Guilford & Dawkins (1991) suggested that there are two crucial conditions to maximize signal effectiveness: that signals should be easily distinguished from each other, and easily detectable against their background. Thus, studies that objectively analyse signal diversity are important to establish, for example, if predators base their rejection of aposematic prey on fine details regarding their coloration, or simply because the signal is salient against a background.

The vast amount of signal variation in species such as poison frogs (Silverstone, 1976; Medina *et al*., 2013), or butterflies (Kapan, 2001), has encouraged detailed work testing which factors might influence signal form to explain the diversity in colour patterns of these species (Lynn *et al*., 2005; Speed & Ruxton, 2007; Mallet, 2010; Chouteau & Angers, 2012). These studies have established quantitative methods to evaluate conspicuousness, and the influence of biotic and abiotic factors in determining optimal signals that are effective against predators and attractive to conspecifics (e.g. Siddiqi, 2004). Speed & Ruxton (2007) also determined that signal diversity may be dependent on population size and seasonality, and the conspicuousness of a signal may be further dependent on resource abundance (Speed & Ruxton, 2007; Blount *et al*., 2012; Flores *et al*., 2013). Finally, the costs of producing a signal could influence the diversity of displays in a species. If these costs are low, a range of signals that have the same fitness benefits may arise (Speed & Ruxton, 2007). In spite of the above, both predators and conspecifics have been predicted to be able to discriminate between the different signals (Siddiqi, 2004; Giraldo *et al*., 2008).

Ladybirds beetles (Coccinellidae) are a diverse group of warningly coloured species (Hodek *et al*., 2012). In the United Kingdom, 26 species subjectively exhibit warning coloration and have secondary chemical defences (Tursch *et al*., 1973; Lognay *et al*., 1996; Ware *et al*., 2007). This impressive variation has been studied in terms of its genetic and thermoregulatory effects (Brakefield, 1985), but not often considered in terms of signal conspicuousness and predator vision (Marples, 1993; Marples *et al*., 1994, 1998) [but see (Blount *et al*., 2012; Winters *et al*., 2014)]. Here, we aimed to establish the diversity of ladybird warning signals in some of the most common species occurring in the United Kingdom and determine how variable they are from a predator's point of view. We first reconstructed the phylogeny of these ladybird species to determine whether the genetic relationships among species have shaped the evolution of their colour attributes. Second, we established a measure of signal uniqueness to determine whether colour patterns differ enough to make clear distinctions between species. Finally, using digital photography (Stevens *et al*., 2007) we quantified the conspicuousness of every ladybird species against three main types of backgrounds: their own (where each species is usually found), an average green and an average brown background. We predicted that life history might influence the existence of local adaptations to decrease predation risk, increasing elytra colour contrast against a species’ preferred substrate. Thus, we considered the foraging habits of every species (generalist/specialist, see below) to determine whether this characteristic affects the conspicuousness of a signal.

## Materials and methods

### Study species and sites

We collected 16 different colour variations, corresponding to 13 ladybird species to analyse the diversity of their coloration. These species subjectively represent most of the colour variation in ladybird elytra colours in the UK (Roy & Majerus, 2011). Table 1 shows the species collected and some of their intraspecific colour variation (where applicable), as well as the number of individuals collected and the location of collection. We also included a general description of the habitat where the individuals were found, and a classification of their habitat use (generalist/specialist). To have a reliable representation of a species’ colour, we collected 20 individuals of all species except *Anatis ocellata* (eyed ladybird, 17) and *Calvia quatuordecimguttata* (cream‐spot their, 10) because of their low availability. All individuals were euthanized in a −80 °C freezer after collection to avoid discomfort. Immediately after, the samples were photographed to ensure that there would be no changes in the colour measurements. Previous experiments have shown that there is no change in a ladybird's elytra reflectance within the first 8 h after death [LME d.f. = 4, 233; *F* = 0.358; *P* = 0.555 (Arenas *et al*., 2014)]. In addition to the ladybird individuals, we also collected samples of the plant or substrate where they were found to use as background measurements. The plants collected were as follows: common nettles (*Urtica dioica*) for two‐spot, 14‐spot, seven‐spot and harlequin ladybirds. European larch twigs (*Larix decidua*) were used for the larch ladybird. Sycamore leaves (*Acer pseudoplantatus*) were the background for orange ladybirds. Scots pine (*Pinus sylvestris*) was used as the background for eyed, striped and pine ladybirds. Common ash bark (*Franixus sp*.) was used as the background of cream‐spot ladybird. Samples of soil were used as the background for *Tytthaspis sedecimpunctata* (16‐spot ladybirds). Sea campion leaves (*Silene uniflora*) were the background for *Subcoccinella vigintiquatuorpunctata* (24‐spot ladybirds), and bird's foot trefoil leaves (*Lotus corniculatus*) were the background for *Coccinella undecimpunctata* (11‐spot ladybirds). We collected a minimum of 10 independent samples of each type of background (including that where each ladybird was found) to have enough variation on their colour attributes.

**Table 1:** Species used in this study: Common and scientific names for the species collected, an image representation of their colour patterns, the type of habitat each species prefers, the location of collection and the number of individuals included in the analyses

Common name	Scientific name	Colour pattern	Preferred habitat	Habitat use	Site of collection	No. individuals collected
Seven spot ladybird	*Coccinella septempunclata*	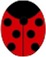	Nettles	Generalist	Cambridge	20
Two spot ladybird.(f. typica)	*Adalia bipunctata*	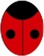	Nettles	Generalist	Cambridge	20
Two spot ladybird (f. melanic)		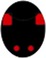	Nettles	Generalist	Falmouth	20
Fourteen spot ladybird	*Propylea quattuordecimpunctata*	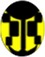	Nettles	Generalist	Cambridge	20
Orange ladybird	*Halyzia sedecimgutatta*	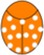	Sycamore	Specialist	Cambridge	20
Striped ladybird	*Myzia oblongoguttata*	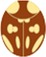	Larch	Specialist	Thetford	20
Harlequin ladybird (f. succinea)		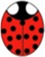				20
Harlequin ladybird (f. conspicua)	*Harmonia axyridis*	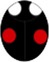	Nettles	Generalist	Cambridge	20
Harlequin ladybird (f. spectabilis)		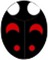				20
Pine ladybird	*Exochomus quadripuslulatus*	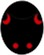	Pine	Specialist	Cambridge	20
Larch ladybird	*Aphidecta obliterata*	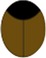	Larch	Specialist	Thetford	20
24‐spot ladybird	*Subcoccinella vigintiquattuorpunctata*	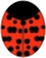	Grasslands and low plants	Specialist	Pendeen	20
Eyed ladybird	*Anatis ocellata*	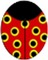	Larch	Specialist	Thetford	17
11‐spot ladybird	*Coccinella undecimpunctata*	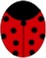	Sand dune plants	Specialist	Newquay	20
16‐spot ladybird	*Tytthaspis sedecimpunctata*	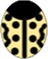	Grasslands and low plants	Specialist	Cambridge	20
Cream‐spot ladybird	*Calvia quattuordecimguttata*	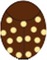	Ash bark	Specialist	Cambridge	10

Larch, eyed, and striped ladybirds and their backgrounds were collected at the King's Forest in Thetford, UK (52°20′17″N, 0°39′58″E). seven‐spot, 14‐spot, two‐spot (*typica*), cream‐spot, harlequin, 16‐spot and pine ladybirds, and background plants were collected in Cambridge, UK (52°12′19.21″N, 0°7′18.54″E). Orange ladybirds and sycamore plants were collected in Madingley Woods, Cambridgeshire, UK (52°13′0.98″N, 0°3′2.93″E). 24‐spot ladybirds and host plants were collected in Pendeen, Cornwall (50°9′51.00″N, 5°40′13.68″W). 11‐spot ladybirds and backgrounds were collected in Hollywell Bay, Cornwall (50°23′22.44″N, 5°8′36.92″W). Two‐spot (*melanic*) ladybirds were kindly provided by Dr. Jon Blount and collected in Falmouth, Cornwall (50°8′33.16′′N, 5°4′13.70′′W).

We used the species descriptions provided by Roy *et al*. (2011) and our own observations during the times of collection (2012–2015), to determine the habitat use of the species collected. A species was classified as a generalist if the number of substrates/plants that it could be found on exceeded three (usually more). This was the case for two‐spot ladybirds (two colour variants), harlequin ladybirds (three colour variants), seven‐spot ladybirds and 14‐spot ladybirds. In contrast, a specialist species would only found on one or two substrates, and were usually harder to find because of the availability of a specific substrate/plant in the wild. Table 1 shows the classification of each species included in this study with respect to its habitat use. In species where there was more than one colour morph included (two‐spot and harlequins), each morph was used as an independent group of data.

### Image collection and set‐up

We used a Nikon D90 digital SLR camera fitted with a AF‐S VR Micro – Nikkor 105‐mm lens, both of which had undergone a UV conversion to enable ultraviolet light to reach the charge‐coupled device (CCD) within the camera (Advanced Camera Services, Norfolk, UK). The photographs were taken under standard conditions in a dark room, with the only light source being a EYE Color Arc ^®^ MT70 bulb (Iwasaki Electric Co. Ltd, Tokyo, Japan). This bulb emits a spectrum similar to a D65 irradiance spectrum (Troscianko & Stevens, 2015), enabling us to have the correct set‐up for UV photography. Each photograph was set up by fixing every individual or piece of background on to a sheet of black ethylene‐vinyl acetate (EVA) used as a low UV‐reflective background (Arenas *et al*., 2014). The camera was fixed at 60 cm from the sample and included a scale, and either one 40% grey standard (Labsphere, Congleton, UK) (samples collected during 2012–2014) or a pair of 8% and 95% polytetrafluoroethylene (PTFE) standards (Troscianko & Stevens, 2015). These standards were used for the calibration of each image, and the difference in reflectance standard did not affect the measurements taken as the calibration takes into account these differences (Troscianko & Stevens, 2015). An ultraviolet and infrared (IR) blocking filter (Baader UV/IR‐Cut/L; 2’’) was used for the visible photographs and includes information from 400 to 700 nm. In addition, we used a visible and IR blocking filter (Baader U; 2’’) which only provides information from 300 to 400 nm.

### Image calibration and analyses

Each image was linearized and normalized according to the camera's sensitivities (Stevens *et al*., 2007) and the grey standards in each photograph. All image calibrations were undertaken using custom‐written plug‐ins for the software Image J (Troscianko & Stevens, 2015). The standards also ensured that we could remove the variation in lighting conditions. We also scaled all images to 16 bits to obtain the maximum amount of information possible from each pixel. We used the reported cone sensitivities for the blue tit [*Cyanistes caeruleus* (Hart *et al*., 2000)] to model each image to a predator's visual space. This process transformed the images we collected into predicted cone catch values using a polynomial mapping technique (Stevens *et al*., 2007; Troscianko & Stevens, 2015). After this transformation, we were able to obtain the ultraviolet (UV), short wave (SW), medium wave (MW), long wave (LW) and double cone (luminance, D) mapped images for both the ladybirds and their backgrounds, which allowed us to measure the cone catch values for each sample in each wavelength. Because ladybird elytra are curved and shiny, their reflectance will be variable depending on the angle that they are viewed from, as a product of specular reflection (Norman *et al*., 2004). Thus, we only measured areas with no specular reflectance to avoid any overrepresentation of the ladybird coloration.

### Phylogenetic comparative analyses

#### Sequence acquisition and alignment

We used the same sequences used by Magro *et al*. (2010) for each species included in this analysis from GenBank (http://www.ncbi.nlm.nih.gov/genbank/). In addition, we downloaded two sequences for the larch ladybird given that this species has not been included in previous studies (accession numbers: HM909101 and KJ963033). We aligned the sequences using C L U S T A L W (Thompson *et al*., 1994) and adjusted this alignment by eye.

#### Phylogenetic reconstruction

We reconstructed the phylogenetic relationships of the species included in this study using three criteria: neighbour joining (NJ), maximum parsimony (MP) and maximum likelihood (ML). The phylogenies were reconstructed in R using the ‘ape’, ‘phangorn’ and ‘phyloch’ packages. For the ML approach, we used RAxML version 7.0.4 (Stamatakis *et al*., 2005) with default settings (GTRGAMMA model). These three methods enabled us to determine how similar the relationships between species were, and choose the best phylogeny to perform further analyses. The trees constructed were indistinguishable from each other. Thus, we chose to use the ML tree for the phylogenetic signal analyses described below.

#### Phylogenetic signal and statistical analyses

Using the R packages ‘phytools’ and ‘picante’, we calculated two separate phylogenetic signal estimators, namely *λ* (Pagel, 1999) and Blomberg's *K* (Blomberg *et al*., 2003) for each of the seven colour attributes measured: elytra hue, elytra saturation, spot luminance, spot saturation, spot hue, internal contrast (spot vs. elytra) and area of the spot covering the elytra. Pagel's *λ* measures phylogenetic dependence in quantitative values (Pagel, 1999; Kamilar & Cooper, 2013). When *λ* approaches zero, traits are said to evolve independently from their relatedness. Blomberg's *K* estimator is estimated as a ratio between the mean square errors (from a GLS model, see below) of the tree tip data in relation to the phylogenetic mean of the data provided. As *K* increases in value, the relationship between the trait and the phylogeny will be less, decreasing the value of the ratio (Revell *et al*., 2008; Kamilar & Cooper, 2013). In addition, we performed a phylogenetic generalized least squares regression (Grafen, 1989) using the R package ‘caper’ using as dependent variable each species’ uniqueness and as independent variables each of the colour measurements mentioned above.

### Signal uniqueness as a measure of diversity

All the species collected subjectively have aposematic coloration; however, because the human perception of coloration is different from that of a predator (Endler, 1978; Endler & Mielke, 2005), these colours might not be perceived as a warning signal by other observers [but see (Ruxton, 2005)]. Using the cone catch values for the UV, SW, MW, LW and D receptors, we measured various aspects of the ladybirds’ colours that could be contributing to the variation in their signals. First, we calculated the saturation of the elytra colour and the spot colour for each individual. Saturation is defined as the perceived intensity of a colour (e.g. red vs. pink). To obtain the saturation values of each individual, we first calculated the proportion of each of the cone‐catch values obtained following the procedures described above. These proportions were used to calculate *X*,* Y* and *Z* Cartesian coordinates of a three‐dimensional tetrahedron representing a tetrachromatic cone‐sensitivity weighted colour space (Endler & Mielke, 2005). Saturation was calculated by determining the Euclidean distance of each point to an achromatic centre of the tetrahedron (with coordinates of *X* = 0, *Y* = 0 and *Z* = 0) (Endler & Mielke, 2005). Second, we measured the spot and elytra hue for each individual. This measurement refers to the type of colour of the signal, and here, it is measured as a ratio of the four colour channels measured (i.e. LW, MW, SW and UV). To calculate hue, we used a previous approach described by Spottiswoode & Stevens (2011) and Stevens *et al*. (2014). This approach uses a principal component analysis (PCA) on a covariance matrix of the standardized photon catches for every wavelength (UV, SW, MW, LW) obtained across individuals. The rationale behind this calculation is that hue is most likely processed by colour opponent mechanisms, which are often defined as a ratios between the cone catch values of each photoreceptor (i.e. in humans Red‐Green = LW/LW + MW or Blue‐Yellow = SW/LW + MW) (Endler, 1990). Although there is some evidence of which opponent mechanisms birds may have evolved (Osorio *et al*., 1999), it is still not certain whether there are further interactions between the photoreceptor types in perceiving colour. Thus, using a PCA it is possible to take into account the eigenvalues that explained most of the variation in this analyses, and calculate a standardized ratio of wavelengths as a measure of the type of colour (Spottiswoode & Stevens, 2011). Note that the colour channels used here do not necessarily mimic any actual opponent colour channels found in avian vision. We calculated two values of hue for the background colours and the ladybird colours, respectively, given that there if often two principal components that explain a large amount of variation (Marshall *et al*., 2015). The ratios used to calculate hue values for the ladybird elytra colours were as follows: (1) H1 = (LW + MW)/(UV + SW) and (2) H2 = (UV + LW)/(MW + SW). We also used the luminance (perceived lightness) of the spots and the elytra of each species, using the double cone (D) value obtained from the photographs. Finally, we measured the internal contrast of the signal (spot against elytra) in luminance (perceived lightness) values. For all of these measurements, we used a log form of the Vorobyev–Osorio model (Vorobyev *et al*., 1998). The model takes into account the sensitivity of each cone in the predator's visual system and estimates of cone abundance and noise in the photoreceptors to calculate discrimination thresholds or ‘just noticeable differences’ (JND). We used the relative cone abundances reported in (Hart *et al*., 2000), combined with a Weber fraction of 0.05 (Vorobyev *et al*., 1998; Hart *et al*., 2000) to establish the noise‐to‐signal ratio (ω_i_) for each receptor. We calculated this value using the formula: *ω*_*i*_ = (0.05/($\eta_{i}^{0.5}$), where *ω*_*i*_ is the noise‐to‐signal ratio for each cone receptor and *η*_*i*_ is the relative cone abundance with respect to the Weber fraction value used. In the model, JND values close to 1.00–3.00 indicate that two objects are likely to be indistinguishable to an observer (avian), and values above this are increasingly likely to lead to detection and discrimination. In this case, because the species we studied are aposematic, we expected the JND values obtained to be well above three JND, giving a measurement of conspicuousness. In addition to the colour attributes of each signal, we also measured the individual's length as an approximation to body size, the area of the elytra and the percentage of area covered by spots on an individual's elytra.

The normality of the variables was determined graphically by analysing the shape of each variable's frequency histograms. All variables were normal except for the JND measurements which were log‐transformed. To analyse signal diversity, we used a discriminant analysis using the package ‘DiscriMiner’ in R Version 3.1.0 ‘Spring Dance’ (R Development Core Team, 2015). We used this package to be able to run a discriminant analysis with a cross‐validation method. This analysis determines how effective the different attributes measured are in separating the data according to a set group classifier [in this case, species (Teasdale *et al*., 2013; Stevens *et al*., 2014)]. In addition, the model generates a predicted model and correlates the classification error to the data collected. The classification matrix compares the predicted model to the real data, and determines how many times each class (species) was misclassified as another, based on a minimum of two attributes. The method for cross‐validation is a ‘leave‐one‐out’ or ‘jackknife’ validation, where parameters are estimated for the total sample and then re‐estimated leaving one of the samples out of the analysis ((Abdi & Williams, 2010). Running a cross‐validated model would also minimize the probability of having type III errors. From the results of this classification analysis, we used the classification matrix to measure the ‘*uniqueness*’ of each species (calculated as the percentage of individuals classified correctly into their class (species). We present the discrimination power of the variables used [correlation ratios (*η* ^2^)], and a Wilk's lambda (*λ*) test to the influence of each variable in discriminating between the classes.

### Intra‐ and interspecific variation in signal conspicuousness and contrast against the background

Our second aim was to determine the level of conspicuousness of different ladybird signals. Given that conspicuousness is most likely to be a measure of the contrast of a signal, rather than the colour *per se* (Stevens & Ruxton, 2012), we analysed this property taking into consideration the background against which the signal is displayed. We photographed the background samples using the same methods described above to obtain cone catch values for each type of background. Using the log form of the Vorobyev‐Osorio model, In addition, we calculated the discrimination thresholds (JNDs) between the colour of the ladybird and the colour of the background. In addition, we calculated a measure of contrast against each background for each species, in terms of colour (using the UV, SW, MW and LW values (Vorobyev *et al*., 1998). Several of the species used in this study exhibit black coloration on their elytra. The perception of very dark black colours is subject to noise from the equipment used, as optical equipment cannot perceive relevant information from colours that have very low reflectance values (J. Troscianko, Personal communication). For this reason, we did not calculate the colour JNDs for the black species, and in their instance, the signal is essentially achromatic anyway.

The normality of the variables was determined by analysing the shape of each variable's frequency histograms. All variables were normal except for the JND measurements which were log‐transformed. We examined the dispersion of our data and the residuals using the LMER Convenience Functions package. The residual plots showed a good distribution of the residuals. To determine whether aposematic signals are more conspicuous against a specific background, we compared the contrast (JNDlog) of each individual (~20) of each ladybird colour (16, corresponding to 13 species) against three types of background: (1) its own (where we collected the samples), (2) an average green background and (3) an average brown background. To calculate the average values, we used the samples of background described above (10 individual collections) to calculate an average green and an average brown cone catch value for each wavelength. Using these average cone catch values, we calculated the JND values between the ladybird samples and each type of background to have a measurement of conspicuousness using the Vorobyev–Osorio model described above (Vorobyev *et al*., 1998). We used the lme4 R package to run linear mixed effect models, with the individual ID as random factor, and the species and contrast type (e.g. own, green, brown) as predicting variables.

In addition, we expected the habitat use (generalist/specialist) of each species to influence ladybird contrast against the three types of background mentioned above. In this context, we expected generalist species to have similar contrast against the three types of backgrounds and specialists to be more contrasting against their own background. To analyse this, we ran an additional linear mixed effect model with the individual ID and species as random factors, and the contrast type (e.g. own, green, brown) as the predicting variable. Markov Chain Monte Carlo (MCMC) *post hoc* comparisons on the relevant variables were carried out using the mcposthoc.fnc function.

## Results

### Phylogenetic comparative analyses

We used the ladybird phylogeny by Magro *et al*. (2010) and additional sequences from GenBank, to build a maximum‐likelihood phylogeny using a RaxML approach (Stamatakis *et al*., 2005) Fig. 1. With this phylogeny, we determined whether the different aspects of the coloration had any association with their phylogenetic relatedness with regard to their uniqueness (Grafen, 1989). We did this by calculating Pagel's *λ* (Pagel, 1999) and Blombergs’ *K* (Blomberg *et al.,* 2003) estimators for phylogenetic signal.

**Figure 1:**
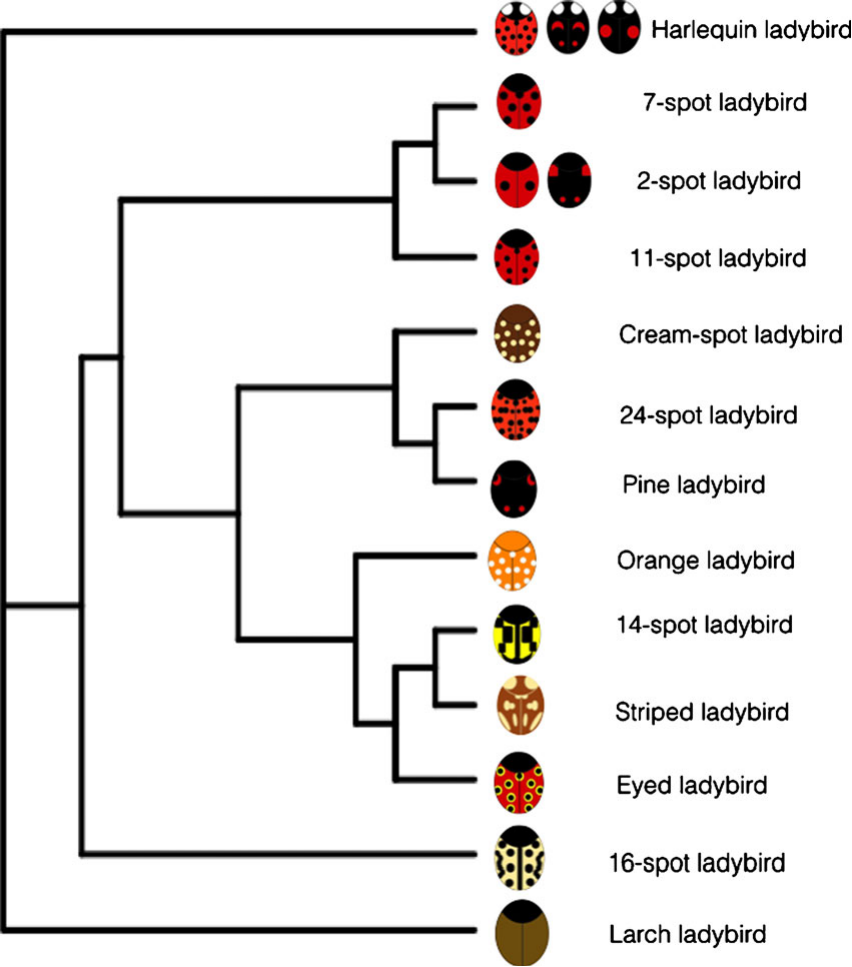
Phylogenetic reconstruction of the ladybird species included in this study using the RaxML algorithm. The phylogenetic signal analyses showed that the relatedness between species is not correlated with attributes of coloration.

In testing for phylogenetic signal, we did not find an influence of relatedness among species in any of the seven colour measurements included in this study. Table 2 shows the detailed results of the phylogenetic regression (PGLS) for every colour attribute. The results of these tests yielded low *λ* values and high *K* values, as well as nonsignificant *P*‐values for both estimators for all the colour measurements included. Furthermore, the phylogenetic regression yielded nonsignificant results, giving further evidence for the lack of association between phylogeny and the for colour attributes measured (i.e. PGLS elytra saturation: d.f. = 1,11; *F* = 0.32; *P* = 0.57). Because the parameters we tested were nonsignificant for the main predicting variables, suggesting no role of phylogeny in the colour parameters tested, we continued our analyses without taking into account the role of phylogeny (Cortesi & Cheney, 2010; Arenas *et al*., 2015).

**Table 2:** Phylogenetic signal analyses: Pagel's lambda (*λ*), Bloomberg's *K* and GLS regression results for the seven colour attributes measured in every ladybird species included in this study

Colour measurement	Phylogenetic signal (*λ*)	Phylogenetic generalised least square regression (pgls)
Elytra hue	*λ *= 0.00; *P* = 1 *K* = 1; *P* = 0.70	*η* ^2^ *P* = 0.56
Elytra saturation	*λ* = 0.00; *P* = 1 *K* = 1; *P* = 1	*η* ^2^ *F* = 0.32; *P* = 0.57
Spot luminance	*λ* = 0.00; *P* = 1 *K* = 1; *P* = 0.76	*η* ^2^ *F* = 1.08; *P* = 0.32
Spot saturation	*λ* = 0.00; *P* = 1 *K* = 1; *P* = 0.71	*η* ^2^ *P* = 0.85
Spot hue	*λ* = 0.00; *P* = 1 *K* = 1; *P* = 0.71	*η* ^2^ *P* = 0.57
Internal contrast	*λ* = 0.00; *P* = 1 *K* = 1; *P* = 0.59	*η* ^2^ *P* = 0.43
Area of spot covering elytra	*λ* = 0.00; *P* = 1 *K* = 1; *P* = 0.72	*η* ^2^ *F* = 0.001; *P* = 0.97

### Signal uniqueness as a measure of diversity

**Figure 2:**
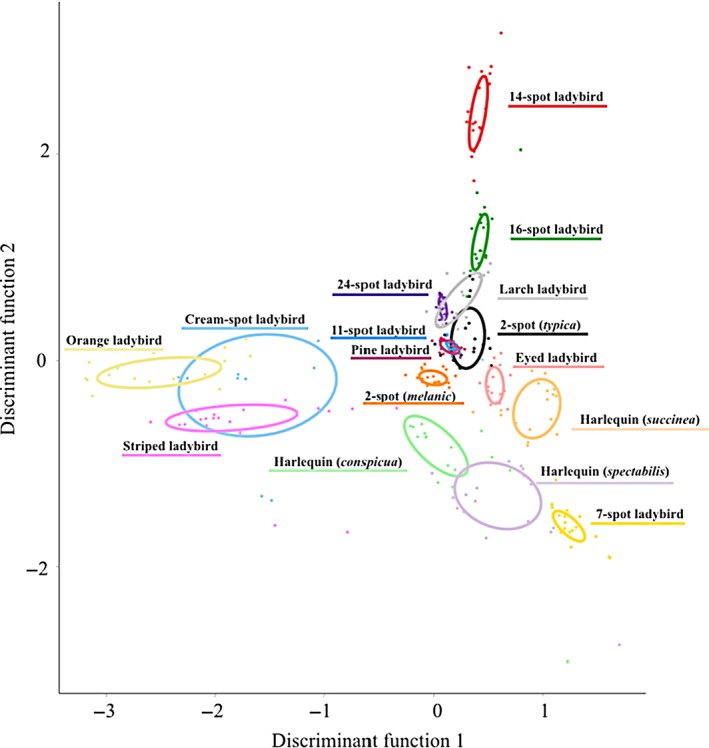
Discriminant plot for nine colour attributes of ladybird coloration. The ellipse around each species represents the 50% of the distribution measuring the Euclidean distance between the centre and every point of each species.

We combined several attributes of ladybird coloration in a cross‐validated discriminant analysis and used the percentage of misclassification of the analysis as a measure of species uniqueness. We found that four of the seven colour variables measured had high discriminating values. Spot luminance (correlation ratio (*η* ^2^) = 0.933; *F* = 261.14; Wilk's *λ* = 0.066; *P* < 0.001), elytra saturation (*η* ^2^ = 0.89; *F* = 154.98; Wilk's *λ* =  0.107; *P* < 0.001), internal contrast (spot vs. elytra contrast) (*η* ^2^ = 0.88; *F* = 137.395; Wilk's *λ* = 0.119; *P* < 0.001) and species size (length) (*η* ^2^ = 0.80; *F* = 78.869; Wilk's *λ* = 0.191; *P* < 0.001) were the variables with the highest discriminant power (Fig. 2). The percentage of correct classification, used as a measure of species uniqueness, revealed that each species is statistically distinguishable from other. Table 3 shows the uniqueness scores (percentage classified correctly) for every species. Figure S1 shows how generalist species (green) and specialists (pink) are distributed in this discriminant space, separating them according to the characteristics included in our analyses.

**Table 3:** Species uniqueness: Percentage of individuals classified correctly into each class (species), based on a cross‐validated discriminant analysis

Species	Appearance	Uniqueness (% ind. classified correctly)
Two‐spot ladybird (*melanic*)	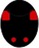	100
Eyed ladybird	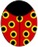	100
11‐spot ladybird	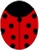	100
14‐spot ladybird	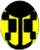	95
Orange ladybird	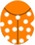	95
Pine ladybird	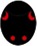	95
16‐spot ladybird	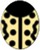	95
Seven‐spot ladybird	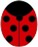	94.4
Striped ladybird	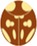	90
Cream‐spot ladybird	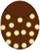	88.8
Harlequin ladybird (*spectabilis*)	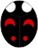	88.3
Harlequin ladybird (*conspicua*)	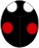	88.2
Larch ladybird	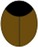	85
24‐spot ladybird	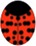	85
Harlequin ladybird (*succinea*)	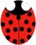	77.7
Two‐spot ladybird (*typica*)	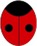	55

### Intra‐ and interspecific variation in signal contrast against the background

#### Species contrast against the background

**Figure 3:**
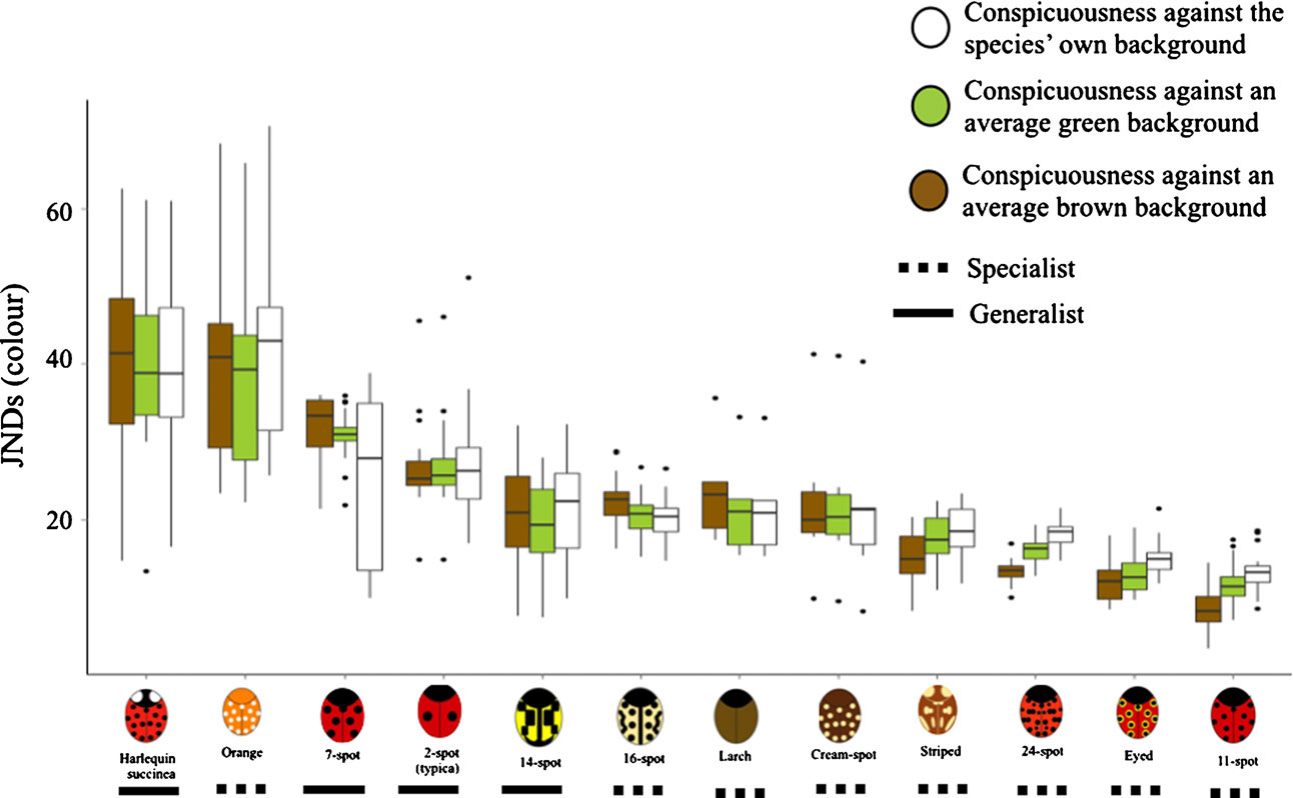
Species conspicuousness (colour contrast) against their own background (where they were collected – white), an average green background (green) and an average brown background (brown). There are significant differences in conspicuousness related to the species and the type of background analysed.

To predict how conspicuous ladybird colours are to a potential avian predator, we calculated the contrast (JNDs) of every individual of every species against three types of background, namely their own (collection site), an average green and an average brown background. Our results suggest that the species differ in their conspicuousness (LME, d.f. = 22, 543; *F* = 17,175; lower *P* < 0.001; Fig. 3). Table 4 shows the detailed *post hoc* conspicuousness comparisons between the three types of background. These tests revealed that the contrast against brown backgrounds is significantly greater than against both green and the species’ own background (*P* < 0.001 for all comparisons). However, the contrast against the average green background and the species’ own background was above 20 JNDs, indicating that the ladybird signals are highly contrasting in a variety of natural backgrounds.

**Table 4:** Conspicuousness against the background: *Post hoc* comparisons results of species conspicuousness analyses against the three background types. Bold values indicate significant differences between the treatments

Contrast type	*Post‐hoc* comparison results
Green vs. brown	d.f. = 576, 371, *t* = −3.347; *P* < 0.001
Green vs. own	d.f. = 576, 371, *t* = 5.955; *P* < 0.001
Brown vs. own	d.f. = 576, 371, *t* = 2.067; *P* < 0.001

#### Conspicuousness and habitat use

**Figure 4:**
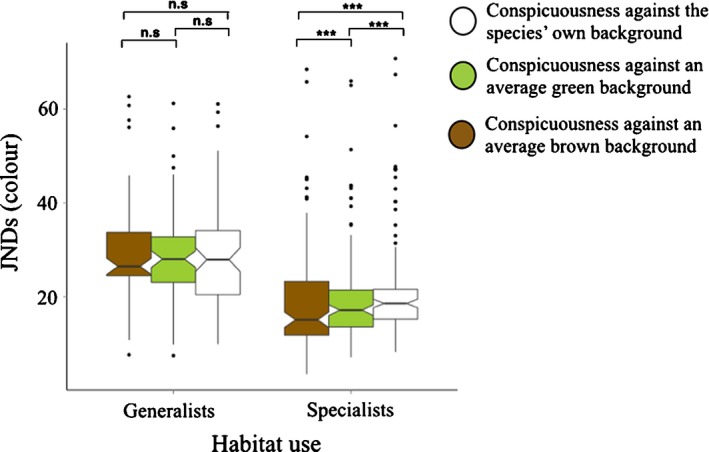
Conspicuousness (colour contrast) is affected by habitat use (generalists vs. specialists). The notch on the boxplots represents the 95% confidence interval of each set of data.

In addition to the differences in species conspicuousness, we also predicted that the differences in habitat use would have an effect on a species’ contrast. We found that, according to our predictions, habitat use is also a good predictor of a species’ conspicuousness (LME, Habitat use*Contrast: d.f. = 2, 573; *F* = 28.63; lower *P* < 0.001). In addition, generalists are more contrasting than specialist species (LME, d.f. = 2, 490; *t* = −4.020; lower *P* < 0.001; Fig. 4). Table 5 shows the detailed results of the *post hoc* tests for the comparisons between the three types of background contrasts analysed. We found that generalists are equally conspicuous against the three types of backgrounds (*P* > 0.05 for all colour comparisons), whereas specialists are significantly more contrasting against their own background.

**Table 5:** Conspicuousness and habitat use: *Post hoc* comparisons results of the species conspicuousness with respect to habitat use analyses. Values in bold show the significant *post hoc* comparisons that yielded significant results

Habitat use	Contrast type	*Post‐hoc* comparison results
Generalist	Green vs. brown	d.f. = 573, 370, *t* = 1.19; *P* = 0.232
	Green vs. own	d.f. = 573, 370, *t* = −1.35; *P* = 0.176
	Brown vs. own	d.f. = 573, 370, *t* = −1.40; *P* = 0.160
Specialist	Green vs. brown	d.f. = 573, 370, *t* = 4.00; *P* < 0.005
	Green vs. own	d.f. = 573, 370, *t* = −3.55; *P* < 0.001
	Brown vs. own	d.f. = 573, 370, *t* = −4.97; *P* < 0.001

## Discussion

In this study, we analysed how diverse ladybird warning signals are, taking into account a predator's (avian) point of view. We measured several aspects of ladybird coloration and pattern to calculate how unique each species’ aposematic pattern is. Further, we calculated the contrast between individuals of every species and natural backgrounds using just noticeable differences (JNDs) as measure of conspicuousness. We did not find any association between the relatedness of the species and their colour patterns. This is perhaps unsurprising, given the strong genetic links in the family (Magro *et al*., 2010). In addition, although phylogeny does not explain clear patterns about habitat use [feeding habits (Magro *et al*., 2010)], these relationships could be more important than coloration. Our results should be interpreted with caution, as the number of species used in the analyses may not be enough to reveal associations between the species due to their colour attributes (Freckleton *et al*., 2002). However, our results coincide with Cortesi & Cheney (2010) and Arenas *et al*. (2015), showing little phylogenetic signal in different measures of coloration of marine opisthobranchs and ladybirds.

Our results suggest that ladybird signals are highly variable and may be easily distinguishable from each other by avian predators, despite how similar some species may look to a human observer (Endler, 1978; Stevens *et al*., 2007). Furthermore, this study uses several characteristics of aposematic signals (not just the colour) to present are an accurate quantitative measurement of the conspicuousness and statistical differences of ladybird colour signals to the vision of avian predators. We found that all species are highly conspicuous against their own, an average green and an average brown background. This shows that ladybird colours are not fully tuned to species‐specific backgrounds, but instead, they seem to provide effective signals overall against a wide range of background types. However, more specifically, we found that specialists are more contrasting against their own background, whereas generalists, although highly conspicuous, are equally contrasting against a variety of backgrounds. This might indicate that life history traits and environmental factors may have influenced habitat use in these animals or that signal form has undergone tuning over evolution in some species in line with their use (Speed & Ruxton, 2007; Arenas *et al*., 2015). Thus, colour signals should be interpreted in line with the specific contexts they are used in (Endler & Day, 2006). For example, male fireflies signalling early in the evening produce yellower bioluminescence that contrasts with high grasses, whereas later in the evening they produce greener lights that contrast more with the environmental light (Hall *et al*., 2016). In addition, Endler and Day (2006) found that bowerbirds tend to use ornaments in their bowers that have more contrasting colours than those of the surrounding area, giving them an advantage in female mate choice.

Signal diversity is difficult to explain because predator education is predicted to be easier if warning signals are monomorphic (Endler, 1988). However, Guilford & Dawkins (1991) argue that for signals to be effective, they must be different from the background habitat in which they are transmitted and seen against, and importantly, distinguishable from other similar signals in the environment (Guilford & Dawkins, 1991; Stevens, 2013). Here, we show that from a predator's point of view ladybird signal variation is large, and each signal is statistically distinguishable from each other. This may imply that ladybird signals are highly effective in communicating unprofitability, although the predator community has to learn a wide variety of patterns, contradicting previous theoretical work (Endler, 1990; Mappes *et al*., 2005). The large amount of signal variation also implies that the chances of encountering the same type of signal twice are relatively low (Endler, 1978). This could favour signal diversity because predation risk would be spread across species resulting in low selection pressures for each colour morph (Endler, 1978; Endler & Greenwood, 1988). The species included in this study have wide overlapping distribution ranges across the UK (Roy *et al*., 2011), supporting our idea that even though the variation may be large, a predator could encounter several aposematic patterns while foraging. Although foraging behaviour may depend on the natural history of the predator and its specific habits (Porter & Labisky, 1986; Naef‐Daenzer, 2000), it is also important to consider which parts of the territory are explored according to prey fluctuation patterns (Robinson & Holmes, 1982).

Another relatively unknown factor is how predators generalize their responses to different warning signals and the role of learning and foraging decisions in determining predator responses (Aronsson & Gamberale‐Stille, 2008; Dolenska *et al*., 2009; Stuckert *et al*., 2013; Skelhorn *et al*., 2016).Previous studies have found that warning signals are rapidly learned, and avoided. However, although using a real predator (birds), these studies tend not test the visual pathways and mechanisms that determine how the signal itself is being perceived and identified. Future studies should assess how signals are processed by the predator's visual and cognitive systems and linked with previous experiences, to promote learning and generalization. We suggest that in addition to colour and pattern characteristics, which have previously been suggested as an important factor to promote learning (Aronsson & Gamberale‐Stille, 2008), signal contrast against the background may also determine the speed at which a specific pattern is learnt and avoided (Endler, 1992). Several studies using real prey species including ladybirds (Arenas *et al*., 2014, 2015), marine invertebrates (Cortesi & Cheney, 2010), milkweed bugs (Prudic *et al*., 2006) and seed bugs (Gamberale‐Stille, 2001) have found that background contrast is important for predator detection (but see Hegna *et al*., 2011 and Darst *et al*., 2006). Furthermore, these results have also been confirmed by studies using artificial stimuli (Osorio *et al*., 1999; Gamberale‐Stille & Guilford, 2003; Arenas *et al*., 2015). These previous findings along with the ones we present here which include a thorough analysis of different characteristics of the signal may be useful for future researchers to infer global trends in the use of aposematic coloration and its evolution.

Our results show that warning signals are highly contrasting with the background, and this could be driving the patterns measured in previous studies, as a general characteristic used for avoidance. It is then crucial to determine which specific and whether other characteristics of the signal can also alter the preferences of a predator. We combined several aspects of each signal displayed by ladybirds, namely colour, pattern and signalling background in an attempt to analyse their coloration in a multidimensional manner (Endler & Day 2006). When taking into account several characteristics of the specific signals, our analyses show that in a multidimensional space, and according to predator visual models, most signals are separated from each other. This could indicate that, despite there being certain boundaries in the divergence of aposematic signals in nature, the convergence of aposematic colours in order to promote fast learning does not mean that all signals need to have the exact colour or only a few colours. Proof of this is the variation of warning colours in species such as poison frogs (Maan & Cummings, 2012), butterflies (Mallet, 2010), ladybirds (Majerus, 2009), nudibranchs (Cortesi & Cheney, 2010) and many others.

High signal variation could also arise as a consequence of local adaptations to specific environments (Mochida, 2011). We found that all species were highly contrasting against a variety of backgrounds, and this could suggest that local adaptations in terms of conspicuousness alone are not the principal factor aiding signal diversity (Prudic *et al*., 2007). However, the life history of many of the species we included in this study is unique, with adaptations to eat certain types of food; for example, the orange ladybird that feeds almost exclusively on mildew (Roy & Majerus, 2011), or the eyed ladybird that prefers aphid species that are abundant in conifers (Kalushkov & Hodek, 2001). Other examples include the restricted habitat distribution of 24‐spot ladybirds, which are only found on low grasses, or the 11‐spot ladybirds, which are restricted to coastal sand dunes (Roy & Majerus, 2011). These characteristics combined with a high detectability in terms of colour and luminance of the signals could be the cause of such diversity in ladybird warning coloration. In this respect, life history, and the interactions between predator vision models and colour patterns, may mean certain morphs could be more prevalent in some environments, even if the number and type of predator remains unchanged (Endler, 1988).

Other factors could also influence the success of warning coloration in nature. Endler (1978) suggested that conspicuous animals benefit from displaying their signals in certain weather conditions, due to shading effects that could illuminate a visual scene in heterogeneous ways. Little is known about how conspicuous coloration is perceived in changing light conditions [but see (Lovell *et al*., 2005) and (Arenas *et al*., 2014)]. Changes in lighting during the day could also affect the way in which a signal is perceived by a predator (Troscianko & Stevens, 2015). In addition, the relationship between a species’ visual signal and the strength of its secondary defences could also affect predator perception and avoidance (Speed & Ruxton, 2007). Finally, signal diversity and effectiveness should be analysed in terms of the cost of producing these signals for aposematic species (Speed & Ruxton, 2007; Lailvaux *et al*., 2012; Zollman *et al*., 2012). Given the success of this strategy in nature, and its role in both sexual advertisements (Maan & Cummings, 2008; Nokelainen *et al*., 2012) and predator avoidance (Maan & Cummings, 2012; Arenas *et al*., 2015), future studies should consider selection pressures and how biotic and abiotic factors shape the evolution of signal diversity and effectiveness in the field.

## Supporting information


**Figure S1** Discriminant plot for the colour attributes of ladybird coloration.
