# Supplementary figures and images for: Diversity in warning coloration is easily recognized by avian predators
Source: J Evol Biol. 2017 Apr 21;30(7):1288–302. doi: 10.1111/jeb.13074 (PMC5518184; doi:10.1111/jeb.13074)

● Generalist species      ● Specialist species

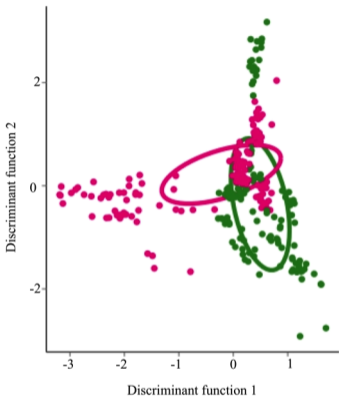

Supplement: Supplementary file 1 — Figure S1 Discriminant plot for the colour attributes of ladybird coloration. [file JEB-30-1288-s001.pdf]
